# The influence of diabetes and antidiabetic medications on the risk of pancreatic cancer: a nationwide population-based study in Korea

**DOI:** 10.1038/s41598-018-27965-2

**Published:** 2018-06-26

**Authors:** Da Young Lee, Ji Hee Yu, Sanghyun Park, Kyungdo Han, Nam Hoon Kim, Hye Jin Yoo, Kyung Mook Choi, Sei Hyun Baik, Nan Hee Kim, Ji A. Seo

**Affiliations:** 1 Division of Endocrinology and Metabolism, Department of Internal Medicine, Korea University College of Medicine, Seoul, Republic of Korea; 2 Department of Biostatics, College of Medicine, The Catholic University of Korea, Seoul, Republic of Korea

## Abstract

This study investigated the effects of diabetes and antidiabetic medications on the risk of pancreatic cancer(PaC). We extracted data on Koreans with newly diagnosed diabetes and selected age- and sex-matched controls provided by the National Health Insurance Corporation. Incident PaC was defined as a new registration in the Korea Central Cancer Registry under ICD-10 C25 with admission history until 2015. During 19,429,617.1 person-years, 8,589 PaCs were identified in 1,005,409 subjects for diabetes group and 4,021,636 subjects for control group. The diabetes group showed more than a two-fold risk for PaC compared with the control group. Among antidiabetic medications, metformin, thiazolidinedione, and dipeptidyl peptidase-4 inhibitor exposure was associated with decreased risk for future PaC(hazard ratio[95% confidence interval] = 0.86[0.77–0.96], 0.82[0.68–0.98], 0.57[0.51–0.64], respectively), whereas sulfonylurea and insulin exposure was related to increased risk(hazard ratio[95% CI] = 1.73[1.57–1.91], 2.86[1.43–5.74], respectively) compared to subjects with no drug exposure. Moreover, subjects with dual exposure history to metformin plus thiazolidinedione or metformin plus dipeptidyl peptidase-4 inhibitor had a lower risk of PaC compared to metformin-only treated subjects. In conclusion, Korean adults with diabetes are at higher risk of PaC compared with nondiabetic individuals, and this risk may be modified by antidiabetic medications.

## Introduction

Pancreatic cancer (PaC) carries a lethal prognosis, and has become the fourth leading cause of cancer mortality worldwide^1^. PaC tends to be diagnosed relatively late and is extremely lethal with a 5-year survival rate of only 9%^1^; therefore, early detection is important. Previous studies have reported several risk factors for PaC, including smoking, obesity, chronic pancreatitis, alcohol consumption, hepatitis B virus infection, family history of PaC, and diabetes^2^.

Diabetes is complexly linked to PaC. Most of PaC patients have either glucose intolerance or diabetes. PaC can cause diabetes, and diabetes is not only thought to be a risk factor for incident PaC^3^, but is also associated with worse treatment outcomes^4^. Furthermore, an association between antidiabetic medication (ADM) and PaC has been reported. Several studies have reported that metformin usage is related to reduced risk of PaC, whereas sulfonylurea and insulin are associated with increased risk^5–7^. In addition, a survival benefit of prior metformin usage in PaC patients with preexisting diabetes has been demonstrated^8^.

Although there are several classes of ADMs, previous significant findings were mainly confined to older medication, including metformin, sulfonylurea, and insulin, whereas data on relatively newer medication such as dipeptidyl peptidase-4 inhibitor (DPP4i) and thiazolidinedione (TZD) are sparse. For DPP4i, a recent elderly cohort study with limited sample size showed lower risk for PaC than for sulfonylurea^9^ and in a large-scale clinical trial, DPP4i-treated subjects showed a numerically but not significantly lower incidence of PaC than placebo-treated subjects^10^.

Recently, the prescription of newer ADMs has steadily increased worldwide including in South Korea because of the lower risk of hypoglycemia and weight gain^11^. Therefore, there is an urgent need for further information about detailed safety profiles^11,12^.

In South Korea, the National Health Insurance Corporation (NHIC) is a health insurance system that is subscribed by approximately 97% of the Korean population and the remaining 3% are covered by a medical aid program. The NHIC holds a huge amount of health information on 50 million Koreans, about eligibility (age, sex, socioeconomic status, type of eligibility, income level, etc.), medical treatment, and health screening examinations, and maintains a medical care institution database (types of medical care institutions, location, equipment and number of physicians)^13^. Additionally, from 2002, the Korea Central Cancer Registry (KCCR) has constructed the Korea National Cancer Incidence Database, with an estimated completeness of 97.8%^14^. These large-scale nationwide population-based data are expected to provide a sufficient number of specific disease cases for analyses.

Hence, the aims of this study are to investigate whether diabetes is related to the risk of PaC in Koreans stratified by age, sex, and observation period, and to evaluate the effects of each class of ADM, in addition to metformin and sulfonylurea, on the risk of PaC.

## Methods

### Data source and selection of study subjects

We retrospectively reviewed the whole health information, provided by the NHIC, of insured Koreans aged ≥30 years. After excluding subjects who were previously diagnosed with diabetes or PaC before December 31, 2008, we included the subjects who were newly diagnosed with diabetes between January 1, 2009, and December 31, 2012, as the diabetes group and selected an age- and sex-matched control group at a 1:4 ratio.

Because healthcare providers submit reports on medical services provided under health insurance policies to the Health Insurance Review and Assessment service for a review of the medical costs incurred in Korea, the medical treatment database is based on medical bills claimed by healthcare providers. Subscribers aged more than 40 years, insured employees or the self-employed are recommended to undergo free standardized medical examinations every two years, which consist of general health examinations including anthropometric and laboratory data (e.g., fasting glucose, lipid profiles, etc.) and questionnaires on lifestyle and behavior.

Study protocols were approved by the official review committee in the NHIC. The study was also reviewed and approved by the Institutional Review Board of the Korea University Ansan Hospital and was carried out in accordance with the Helsinki Declaration of 1975.

### Study outcomes

The endpoints of this study were the first diagnoses of PaC until December 31, 2015. Incident PaC was defined as a new registration in the KCCR under the International Classification of Diseases, 10th edition (ICD-10) C25^15^ with admission histories in hospitals, except for those from dental hospitals, pharmacies, or the Korean Oriental Medicine Clinic. The index date was the date of the first diagnosis of diabetes in the diabetes group and the date of the first health screening exam or January 1, 2009, in the control group. The observation period was calculated using the duration between index date and diagnosis date in PaC patients or December 31, 2015, in none-PaC subjects.

### The operational definition of diabetes and other conditions

The presence of diabetes was determined by the following criteria: (1) the presence of at least one claim per year for the prescription of ADM under ICD-10 E10-14, or (2) fasting glucose level ≥7.0 mmol/L obtained from the health examination database. ADMs were classified into metformin, sulfonylurea, meglitinide, TZD, DPP4i, alpha-glucosidase inhibitor (AGI), and insulin. The glucagon-like peptide-1 receptor agonist was not included in this study because health insurance coverage has only been available since November 2010 with strict conditions^12^. Drug exposure was defined as a prescription history of ≥90 days until one year before the diagnosis of PaC. That is, subjects with a prescription history of 1–89 days were classified as nonusers.

We defined several risk factors for PaC using ICD-10 codes^2^: chronic pancreatitis (K86.0 and K86.1); acute pancreatitis (K85); hepatitis B virus (B16, B18.0, and B18.1), hepatitis C virus (B17.1 and B18.2); biliary diseases including cholecystitis, cholangitis, cholelithiasis, and choledocholithiasis (K80-K83); alcoholism defined as ICD-10 F10.2 or heavy alcohol consumption (≥30 g/day) in the questionnaire obtained from the health examination database; and non-alcoholic fatty liver disease (NAFLD, K76.0).

The presence of hypertension was determined by the presence of at least one claim per year for the prescription of antihypertensive drugs under ICD-10 codes I10-I15, or systolic blood pressure (BP) ≥140 mmHg and diastolic BP ≥90 mmHg obtained from the health examination database. The presence of dyslipidemia was determined by the presence of at least one claim per year for the prescription of antihyperlipidemic agents under ICD-10 codes E78, or total cholesterol levels ≥6.21 mmol/L obtained from the health examination database. Previous history of stroke, transient ischemic attack, and myocardial infarction were defined as the presence of ICD-10 I63-64, G458-459, and I21-22, respectively.

Income status was stratified into quartile and medical aid.

### Variables derived from the health screening exam database

The participants of a government-operated health examination answered a questionnaire addressing demographic characteristics and lifestyle habits, such as smoking status, alcohol consumption, and exercise. Body mass index (BMI) was calculated as weight in kilograms divided by the square of height in meters. BP was measured using a standardized sphygmomanometer after 5 minutes of rest. Venous blood samples were collected in the morning after an overnight fast of at least 8 hours. Serum levels of glucose, total cholesterol, triglycerides, high-density lipoprotein cholesterol, aspartate aminotransferase, alanine aminotransferase, and gamma-glutamyl transferase were measured. Quality control procedures for laboratory tests have been conducted in accordance with the Korean Association of Laboratory Quality Control.

### Statistical analysis

Data are presented as the mean ± standard deviation (SD), geometric mean (95% confidence interval [CI]), or number (percentage). The baseline characteristics were compared between the diabetes and control group using *t*-tests for continuous variables and the chi-squared test for categorical variables.

To assess the relative risk of incident PaC, we performed Cox proportional hazard analyses in the total subjects after stratifying them according to age (30–39, 40–64, and ≥65 years), sex, and observation period (1.1–2.0, 2.1–4.0, and ≥4.1 years) with the control group as a reference. We excluded the subjects whose follow-up period was less than 12 months to reduce the potential for reverse causality.

To evaluate the effect of ADM on PaC risk related to diabetes, we conducted Cox analysis confined to the diabetes group with nonusers of each kind of ADM as a reference. The relative risk of PaC was estimated in the whole diabetes group, including drug-naïve subjects, and in the treated diabetes group with a history of more than 90 days prescription of ADM. Given metformin has been used as a first-class ADM^16^, we examined the relative risk of incident PaC in subjects who have taken other classes of ADM in addition to metformin compared to metformin-only users.

In the above-mentioned Cox analyses, we adjusted for age, sex, chronic pancreatitis, acute pancreatitis, hepatitis B, hepatitis C, biliary disease, alcoholism, NAFLD, income of the lowest quartile, place of residence, and the number of different exposed ADM as confounders. For stricter adjustment of confounders, subgroup analyses were conducted with a selection of subjects who had available health screening exam data at baseline. Fasting serum glucose level, BMI, and current smoking status were additionally adjusted in subgroup analyses. We confirmed the variable inflation factor for all covariates of less than 2.0, indicating no relevant multicollinearity among covariates. SAS version 9.3 (SAS Institute Inc., Cary, NC, USA) was used for statistical analysis. A *p* value of <0.05 was considered to be statistically significant.

### Data availability

The data that support the findings of this study are available from the National Health Insurance Corporation but restrictions apply to the availability of these data, which were used under license for the current study, and so are not publicly available. Data are however available from the authors upon reasonable request and with permission of the National Health Insurance Corporation.

### Ethics approval and consent to participate

All participants provided written informed consent. Study protocols were approved by the official review committee in the NHIC. The study was also reviewed and approved by the Institutional Review Board of the Korea University Ansan Hospital and was carried out in accordance with the Helsinki Declaration of 1975.

## Results

A total of 1,005,409 subjects from the diabetes group and 4,021,636 subjects from the control group were identified for analysis. As shown in Table 1, the proportion of individuals who had a risk factor for PaC and cardiometabolic diseases were higher in the diabetes group than in the control group.

**Table 1:** Baseline characteristics of study subjects according to incident diabetes.

	Control (N = 4,021,636)	Diabetes (N = 1,005,409)	*p* value
Age (years)	57.2 ± 12.5	57.2 ± 12.5	1
30–39	322,396 (8.0)	80,599 (8.0)	
40–64	2,500,100 (62.2)	625,025 (62.2)	
≥65	1,199,140 (29.8)	299,785 (29.8)	
Sex, male (%)	2,236,900 (55.6)	559,225 (55.6)	1
Comorbidities			
Hypertension (%)	1,021,775 (25.4)	523,654 (52.1)	<0.001
Dyslipidemia (%)	490,804 (12.2)	432,661 (43.0)	<0.001
Stroke (%)	137,904 (3.4)	71,141(7.1)	<0.001
Myocardial infarction (%)	27,944 (0.7)	21,921 (2.2)	<0.001
TIA (%)	36,794 (0.9)	18,046 (1.8)	<0.001
Chronic pancreatitis (%)	6,648 (0.2)	8,186 (0.8)	<0.001
Acute pancreatitis (%)	19,825 (0.5)	20,221 (2.0)	<0.001
Hepatitis B (%)	66,873 (1.7)	39,675 (4.0)	<0.001
Hepatitis C (%)	21,822 (0.5)	19,584 (2.0)	<0.001
Biliary disease (%)	53,835 (1.3)	36,706 (3.7)	<0.001
Alcoholism (%)	73,946 (1.8)	70,491 (7.0)	<0.001
NAFLD (%)	401,238 (10.0)	333,593 (33.2)	<0.001
Place of residence			<0.001
Urban	1,834,944 (45.6)	448,736 (44.6)	
Rural	2,186,692 (54.4)	556,673 (55.4)	
Income status (%)			<0.001
Quartile 1	960,140 (23.9)	247,883 (24.7)	
Quartile 2	870,293 (21.6)	218,062 (21.7)	
Quartile 3	938,082 (23.3)	227,968 (22.7)	
Quartile 4	1,086,158 (27.0)	247,814 (24.7)	
Medical aid	166,963 (4.2)	63,682 (6.3)	
Year of index date			1
2009	1,009,616 (25.1)	252,404 (25.1)	
2010	994,300 (24.7)	248,575 (24.7)	
2011	1,042,652 (25.9)	260,663 (25.9)	
2012	975,068 (24.3)	243,767 (24.3)	

Data are presented as mean ± standard deviation, or number (%).

The Student’s *t*-test or chi-squared test was used to compare the characteristics of the study participants at baseline.

Abbreviations: TIA, transient ischemic attack; NAFLD, non-alcoholic fatty liver disease.

During 19,429,617.1 person-years of follow-up (median [interquartile range] of follow-up period was 4.9 [3.9–6.0] years), 8,589 new cases of PaC were identified from the KCCR after excluding subjects whose follow-up period was less than 12 months. The diabetes group had more than twice the risk of incident PaC compared with the control group (Table 2). When we stratified subjects according to age, sex, and observation period, this significance was consistently observed in all groups after adjustment for the risk factors of PaC.

**Table 2:** Risk of incident pancreatic cancer in total subjects and according to sex, age, and observation period.

	Number of subjects	Number of events	Follow-up period	Incidence rate	Multivariate-adjusted HR^*^ (95% CI)	
					Model 1	Model 2
Total	4,945,886	8,589	19,429,617.1	0.44		
Control	3,979,394	5,673	15,673,239.8	0.36	1 (ref.)	1 (ref.)
Diabetes	966,492	2,916	3,756,377.3	0.78	2.22 (2.12–2.32)	2.06 (1.97–2.16)
**Age (years)**						
30–39						
Control	322,059	30	1,295,189.3	0.02	1 (ref.)	1 (ref.)
Diabetes	79,737	25	318,619.1	0.08	3.39 (2.00–5.77)	2.75 (1.51–4.99)
40–64						
Control	2,490,209	2,130	9,920,518.6	0.21	1 (ref.)	1 (ref.)
Diabetes	611,158	1,255	2,406,050.7	0.52	2.45 (2.28–2.62)	2.22 (2.07–2.39)
≥65						
Control	1,167,126	3,513	4,457,531.8	0.79	1 (ref.)	1 (ref.)
Diabetes	275,597	1,636	1,031,707.4	1.59	2.04 (1.93–2.17)	1.94 (1.83–2.07)
Male						
Control	2,211,831	3,395	8,685,416.1	0.39	1 (ref.)	1 (ref.)
Diabetes	536,230	1,727	2,071,722.4	0.83	2.21 (2.09–2.34)	2.02 (1.90–2.15)
Female						
Control	1,767,563	2,278	6,987,823.7	0.33	1 (ref.)	1 (ref.)
Diabetes	430,262	1,189	1,684,654.9	0.71	2.22 (2.07–2.38)	2.12 (1.97–2.28)
**Observation period (years)**						
1.1–2.0						
Control	42,694	1,210	21,349.7	56.68	1 (ref.)	1 (ref.)
Diabetes	17,235	841	8,365.1	100.54	1.65 (1.51–1.81)	1.70 (1.55–1.86)
2.1–4.0						
Control	1,020,866	2,564	2,532,590.2	1.01	1 (ref.)	1 (ref.)
Diabetes	253,402	1,308	623,532.7	2.10	2.14 (2.00–2.29)	2.06 (1.92–2.21)
≥4.1						
Control	2,915,834	1,899	13,119,299.9	0.15	1 (ref.)	1 (ref.)
Diabetes	695,855	767	3,124,479.5	0.25	1.76 (1.62–1.91)	1.65 (1.51–1.80)

Cox proportional hazard models were used to estimate hazard ratios and 95% confidence intervals.

Model 1 was adjusted for age and sex. Model 2 is the same as model 1 plus an adjustment for chronic pancreatitis, acute pancreatitis, hepatitis B, hepatitis C, biliary disease, alcoholism, non-alcoholic fatty liver disease, income of lowest quartile, and place of residence.

HR, hazard ratio; CI, confidence interval.

In Cox analysis conducted only in the diabetes group, subjects with metformin, TZD, and DPP4i exposure showed a significantly lower risk of PaC, whereas sulfonylurea and insulin usage was associated with a higher risk of PaC (Table 3) compared to those that took no drugs, after adjustment for age, sex, history of morbidities associated with the risk of PaC, and socioeconomic status. AGI usage was related to an increased risk of PaC with further adjustment for the number of different exposed ADM.

**Table 3:** Risk of incident pancreatic cancer according to drug exposure in the diabetes group.

	Number of subjects	Number of events	Follow-up period	Incidence rate	Duration of diabetes	Number of exposed ADM	Multivariate-adjusted HR^*^ (95% CI)		
							Model 1	Model 2	Model 3
Non-metformin	277,797	1,076	1,021,837.2	1.05	3.7	0.3	1 (ref.)	1 (ref.)	1 (ref.)
Metformin	688,656	1,839	2,734,540.1	0.67	4.0	2.1	0.75 (0.70–0.81)	0.79 (0.73–0.85)	0.86 (0.77–0.96)
Non-sulfonylurea	591,227	1,629	2,184,190.8	0.75	3.7	1.0	1 (ref.)	1 (ref.)	1 (ref.)
Sulfonylurea	375,226	1,286	1,572,186.5	0.82	4.2	2.5	1.16 (1.08–1.25)	1.19 (1.10–1.28)	1.73 (1.57–1.91)
Non-meglitinide	955,615	2,881	3,708,855.1	0.78	3.9	1.6	1 (ref.)	1 (ref.)	1 (ref.)
Meglitinide	10,838	34	47,522.2	0.72	4.4	2.8	0.81 (0.56–1.14)	0.78 (0.60–1.10)	0.88 (0.63–1.24)
Non-TZD	902,728	2,786	3,479,655.1	0.80	3.9	1.5	1 (ref.)	1 (ref.)	1 (ref.)
TZD	63,725	129	276,722.2	0.47	4.3	3.1	0.70 (0.59–0.84)	0.71 (0.60–0.85)	0.82 (0.68–0.98)
Non-DPP4i	622,982	2,283	2,366,181.4	0.96	3.8	1.0	1 (ref.)	1 (ref.)	1 (ref.)
DPP4i	343,471	632	1,390,195.9	0.45	4.0	2.6	0.60 (0.55–0.65)	0.61 (0.56–0.66)	0.57 (0.51–0.64)
Non-AGI	921,298	2,735	3,553,743.7	0.77	3.9	1.5	1 (ref.)	1 (ref.)	1 (ref.)
AGI	45,155	180	202,633.6	0.89	4.5	3.0	1.09 (0.94–1.27)	1.09 (0.93–1.26)	1.29 (1.10–1.51)
Non-insulin	965,327	2,907	3,751,570.0	0.77	3.9	1.6	1 (ref.)	1 (ref.)	1 (ref.)
Insulin	1,126	8	4,807.4	1.66	4.3	2.9	2.93 (1.47–5.87)	2.52 (1.26–5.04)	2.86 (1.43–5.74)

Cox proportional hazard models were used to estimate hazard ratios and 95%confidence intervals.

Model 1 was adjusted for age and sex. Model 2 is the same as model 1 plus an adjustment for chronic pancreatitis, acute pancreatitis, hepatitis B, hepatitis C, biliary disease, alcoholism, non-alcoholic fatty liver disease, income of lowest quartile, and place of residence. Model 3 is the same as model 2 plus an adjustment for the number of different exposed anti-diabetic medications.

No, number; ADM, anti-diabetic medication; HR, hazard ratio; CI, confidence interval; AGI, alpha-glucosidase inhibitor; TZD, thiazolidinedione; DPP4i, dipeptidyl peptidase-4 inhibitor; AGI, alpha-glucosidase inhibitor.

We repeated the analyses confined to subjects who have ≥90 days of prescription histories of more than one type of ADM (Supplementary Table S1). Similar protective effects of TZD and DPP4i and harmful effects of sulfonylurea and insulin on the risk of PaC were observed. After full adjustment including the number of different ADM exposures, the protective effect of TZD exposure disappeared.

When we confined Cox analyses to metformin users, dual exposure to TZD or DPP4i plus metformin led to a significantly decreased risk of PaC, whereas sulfonylurea or insulin plus metformin were related to increased risk of PaC compared to metformin-only exposure (Table 4).

**Table 4:** Risk of incident pancreatic cancer according to drug exposure in metformin users.

	Number of subjects	Number of events	Follow-up period	Incidence rate	Duration of diabetes	Multivariate-adjusted HR^*^ (95% CI)	
						Model 1	Model 2
Metformin only vs.	177,590	455	645,893.2	0.70	3.6	1 (ref.)	1 (ref.)
+sulfonylurea	320,457	995	1,351,364.0	0.74	4.2	1.26 (1.12–1.41)	1.25 (1.12–1.40)
+meglitinide	7,630	21	34,895.1	0.70	4.6	0.89 (0.57–1.38)	0.87 (0.56–1.34)
+TZD	57,484	88	250,460.1	0.70	4.4	0.66 (0.53–0.84)	0.66 (0.52–0.84)
+DPP4i	330,839	612	1,343,206.8	1.71	4.1	0.85 (0.75–0.96)	0.85 (0.75–0.96)
+AGI	36,395	131	166,896.9	0.70	4.6	1.23 (1.01–1.50)	1.20 (0.99–1.46)
+insulin	798	6	3,517.5	0.60	4.4	3.80 (1.69–8.54)	3.19 (1.41–7.24)

Cox proportional hazard models were used to estimate hazard ratios and 95% confidence intervals.

Model 1 was adjusted for age and sex. Model 2 is the same as model 1 plus an adjustment for chronic pancreatitis, acute pancreatitis, hepatitis B, hepatitis C, biliary disease, alcoholism, non-alcoholic fatty liver disease, income of lowest quartile, and place of residence.

No, number; IR, incidence rate; ADM, anti-diabetic medication; HR, hazard ratio; CI, confidence interval; AGI, alpha-glucosidase inhibitor.

For subgroup analysis, 864,949 subjects from the diabetes group and 3,459,796 subjects from the control group who had available health examination data were selected. A total of 5,225 cases of PaC developed in these subgroups after excluding subjects whose follow-up period was less than 12 months. The diabetes group showed worse metabolic profiles than the control group (Supplementary Table S2). When we additionally adjusted fasting serum glucose levels at baseline, BMI, and current smoking as confounders, the diabetes group still showed a significantly higher risk of incident PaC compared with the control group, especially in those aged more than 40 years and whose observation period was more than two years (Supplementary Table S3). In subjects who had a history of ADM exposure, metformin and DPP4i exposure were associated with a decreased risk of PaC, whereas sulfonylurea exposure was correlated with increased risk (Supplementary Table S4) compared to those who were not exposed to ADMs. In metformin users, exposure to both metformin and TZD or metformin and DPP4i led to decreased risk for PaC versus metformin-only exposure (Supplementary Table S5).

## Discussion

To the best of our knowledge, this is the largest nationwide population-based cohort study that has shown an increased risk of incident PaC in diabetes patients regardless of age, sex, and observation period. In this study, not only metformin but also DPP4i or TZD exposure was associated with decreased risk of future PaC, whereas sulfonylurea or insulin exposure increased the risk. In addition, subjects with dual exposure to metformin plus TZD or metformin plus DPP4i were at lower risk of PaC compared with metformin-only treated subjects.

The present study confirms the previous findings of several population-based studies regarding the elevated risk for PaC in diabetic patients^17,18^. However, in those studies, a significant relationship was observed only in patients aged over 45 years^17^, or in those with a diabetic duration of less than 2 years^18^. More consistent results might have been obtained across age groups and disease durations by using a sufficient number of study subjects and PaC cases.

Numerous studies have examined whether ADM might modify the risk of PaC^7^. Our findings are consistent with previous studies that have reported risk reduction in incident PaC with metformin^5,6^ and risk increase with sulfonylurea exposure^5–7^. The antitumor effects of metformin can be mediated by direct action on the adenosine monophosphate-activated protein kinase signaling pathway and the consequent inhibition of the mammalian target of the rapamycin pathway, as well as secondary effects on insulin sensitivity and hyperinsulinemia^19^. Sulfonylurea is thought to promote carcinogenesis by increasing insulin-like growth factor-1 (IGF-1) activity, resulting in the abnormal stimulation of various cellular signaling cascades, enhancing growth factor-dependent cell proliferation, and affecting cell metabolism^19^.

One of the novel findings of the present study is the cancer-protective implication of DPP4i. Its impact on PaC risk has not yet been conclusively demonstrated. Analyses from the adverse event reporting system of the US Food and Drug Administration and the National Health Insurance database of Taiwan suggested a causal relationship between sitagliptin usage and the risk of PaC^20,21^. However, in a USA study of Medicare claims data evaluating cardiovascular outcomes with sitagliptin, there was no significant effects on PaC^9,10^. A common limitation of the aforementioned studies was an insufficient number of PaC cases due to its low incidence rate^9,20,21^. Although DPP4i has favorable safety profiles, such as a low risk of hypoglycemia or weight gain, and effectiveness in glycemic control, the high price is a stumbling block for its usage^16,22^. In Korea, however, the use of DPP4i has dramatically increased since 2009, and comprised one-third of the market share in 2013 because of the approval for reimbursement, with metformin dominating the market over this period^11,12^. Therefore, we could include a high incidence of DPP4i usage in our study. Additionally, because the data from NHIC and KCCR covered the whole population of Korea, we obtained sufficient PaC cases for analysis, and simultaneous assessment of the effects of ADMs on PaC was possible. A preclinical study suggested that the antitumor effect of DPP4i is due to downregulation of autophagy, increased apoptosis, and cell cycle arrest^23^. Given that DPP4i has been widely used as a second-line therapy after metformin^16,22^, most DPP4i users were also metformin users. To avoid overestimating the protective effect of DPP4i by a possible combined metformin antitumor effect, we repeated the analyses confined to metformin users. Interestingly, dual exposure to DPP4i and metformin was more protective against PaC than metformin-only exposure. Further studies on this possible synergistic effect will be needed.

This study showed the protective impact of TZD exposure on the risk of PaC for the first time. Previous population-based studies in diabetes showed the association of TZD usage with decreased risk of liver cancer^24,25^, but not of PaC^7,18^. However, again, the number of PaC cases in those studies was too small to reach statistical significance. The antitumor effect of TZD has already been suggested by several researchers^24–26^. Galli *et al*. reported that TZD could inhibit cell growth and induce ductal differentiation by peroxisome proliferator-activated receptor γ (PPARγ) pathways, and the PPARγ-independent pathway could inhibit cell invasiveness by the inhibition of gelatinolytic and fibrinolytic activity^26^.

An increased risk for PaC has been suggested in cases of insulin-based therapies^6^. Because subcutaneous injection of insulin might induce higher levels of systemic insulin than endogenous insulin secretion, insulin therapy could amplify the possible relationship between hyperinsulinemia and malignancy risk by excessive insulin binding to the IGF-I receptor^27^. All of the diabetic subjects in this study were newly diagnosed; therefore, only 1% of subjects in the diabetes group were exposed to insulin and very few cases of PaC (8 cases) developed. Nevertheless, a significantly increased hazard ratio (HR) with wide CIs was found in the subjects with insulin exposure.

The results of AGI usage should be interpreted cautiously. Through enhancing colonic butyrate production, thereby possibly promoting colonic differentiation and nutrition, AGI is thought to be associated with a low risk of gastrointestinal cancer^28^. However, conflicting results in cancer incidence were also observed in previous meta-analysis by Wu *et al*., which showed that AGI usage was associated with a 10% increased risk of cancer incidence^29^. In this study, AGI users showed increased HRs compared to nonusers or metformin-only users in some models, but no significant effects were observed in models fully adjusted for glucose, obesity, and smoking status in subgroup analyses. Also, AGI users occupied a relatively small portion of subjects in our study. A future prospective study on this topic is needed.

Despite several strengths, this study has limitations. First, reverse causality is a major concern. Because PaC is a diabetogenic state in itself, diabetes patients with PaC might experience rapid worsening of glycemic status, thereby switching or adding ADMs. Thus, we excluded PaC events that occurred within one year after inclusion to minimize reverse causality, and defined drug exposure as a prescription dating to more than one year prior to the diagnosis of PaC. When we excluded the subjects whose follow-up period less than two years, these significances were consistently observed (Supplementary Table S6). Second, the dose of each drug, medication compliance, or exact glycemic control status could not be considered in this large epidemiologic study. However, a randomized clinical trial assessing a specific ADM’s effects on the incidence of PaC is very difficult to realize because of the required sample size and duration of follow-up^30^. We tried to consider the degree of hyperglycemia by adjusting the different types of ADM exposure and fasting glucose level at baseline in subgroup analyses. Third, the long-term effects of each ADM could not be investigated owing to the relatively short average follow-up period of 4.8 years. Finally, the type of diabetes was not differentiated because ICD codes cannot be used to precisely distinguish type 1 and type 2 diabetes. However, we expect the proportion of type 1 diabetes and its impact was negligible because the incidence of PaC in insulin users was very small.

In conclusion, Korean adults with diabetes are at high risk of PaC compared with nondiabetic individuals and the degree of risk varies among ADM users. Metformin, DPP4i or TZD exposure is related to decreased risk for the development of PaC, while sulfonylurea or insulin exposure is associated with increased risk. The findings from this nationwide cohort could provide clinically meaningful evidence for the emphasized screening of PaC in diabetic patients. Further studies with longer follow-up and in other ethnicities are required to confirm our results about the effects of each ADM class on the risk of PaC.

## Electronic supplementary material

Supplementary Table S1-S6
