# Supplementary material for: The influence of diabetes and antidiabetic medications on the risk of pancreatic cancer: a nationwide population-based study in Korea
Source: Sci Rep. 2018 Jun 26;8:9719. doi: 10.1038/s41598-018-27965-2 (PMC6018762; doi:10.1038/s41598-018-27965-2)
Supplement: Supplementary file 1 — Supplementary Table S1-S6 [file 41598_2018_27965_MOESM1_ESM.docx]

**The influence of diabetes and antidiabetic medications on the risk of pancreatic cancer: a nationwide population-based study in Korea**

Da Young Lee^1^, Ji Hee Yu^1^, Sanghyun Park^2^, Kyungdo Han^2^, Nam Hoon Kim^1^, Hye Jin Yoo^1^, Kyung Mook Choi^1^, Sei Hyun Baik^1^, Nan Hee Kim^1^, Ji A Seo^1^

^1^Division of Endocrinology and Metabolism, Department of Internal Medicine, Korea University College of Medicine, Seoul, Republic of Korea

^2^ Department of Medical Statistics, College of Medicine, The Catholic University of Korea, Seoul, Republic of Korea

|  | Number of  subjects | Number of  events | Follow-up period | Incidence rate | Duration of diabetes | Number of  Exposed ADM | Multivariate-adjusted HR^*^ (95% CI) | | |
| --- | --- | --- | --- | --- | --- | --- | --- | --- | --- |
|  |  |  |  |  |  |  | Model 1 | Model 2 | Model 3 |
| Non-metformin | 67,478 | 341 | 267,463.2 | 1.27 | 4.0 | 1.3 | 1(ref.) | 1(ref.) | 1(ref.) |
| Metformin | 688,656 | 1,839 | 2,734,540.1 | 0.67 | 4.0 | 2.1 | 0.72 (0.64-0.80) | 0.73 (0.65-0.82) | 0.77 (0.68-0.87) |
| Non-sulfonylurea | 380,908 | 894 | 1,429,816.8 | 0.63 | 3.8 | 1.6 | 1(ref.) | 1(ref.) | 1(ref.) |
| Sulfonylurea | 375,226 | 1,286 | 1,572,186.5 | 0.82 | 4.2 | 2.5 | 1.35 (1.24-1.47) | 1.34 (1.23-1.47) | 1.69 (1.54-1.87) |
| Non-meglitinide | 745,296 | 2,146 | 2,954,481.1 | 0.73 | 4.0 | 2.0 | 1(ref.) | 1(ref.) | 1(ref.) |
| Meglitinide | 10,838 | 34 | 47,522.2 | 0.72 | 4.4 | 2.8 | 0.83 (0.59-1.17) | 0.80 (0.57-1.13) | 0.87 (0.62-1.23) |
| Non-TZD | 692,409 | 2,051 | 2,725,281.1 | 0.75 | 3.9 | 1.9 | 1(ref.) | 1(ref.) | 1(ref.) |
| TZD | 63,725 | 129 | 276,722.2 | 0.47 | 4.3 | 3.1 | 0.75 (0.62-0.89) | 0.75 (0.62-0.89) | 0.84 (0.69-1.01) |
| Non-DPP4i | 412,663 | 1,548 | 1,611,807.5 | 0.96 | 3.9 | 1.5 | 1(ref.) | 1(ref.) | 1(ref.) |
| DPP4i | 343,471 | 632 | 1,390,195.9 | 0.45 | 4.0 | 2.6 | 0.62 (0.56-0.68) | 0.62 (0.56-0.68) | 0.57 (0.51-0.64) |
| Non-AGI | 710,979 | 2,000 | 2,799,369.7 | 0.71 | 3.9 | 2.0 | 1(ref.) | 1(ref.) | 1(ref.) |
| AGI | 45,155 | 180 | 202,633.6 | 0.89 | 4.5 | 3.0 | 1.14 (0.98-1.32) | 1.12 (0.96-1.31) | 1.29 (1.10-1.51) |
| Non-insulin | 755,008 | 2,172 | 2,997,196.0 | 0.72 | 4.0 | 2.0 | 1(ref.) | 1(ref.) | 1(ref.) |
| Insulin | 1,126 | 8 | 4,807.4 | 1.66 | 4.3 | 2.9 | 3.17 (1.59-6.34) | 2.78 (1.39-5.58) | 3.05 (1.52-6.12) |

**Supplementary Table S1.** Risk of incident pancreatic cancer according to each drug exposure in subjects with more than 90-day prescription histories for more than one type of anti-diabetic medication

Cox proportional hazard models were used to estimate hazard ratios and 95% confidence intervals.

Model 1 was adjusted for age and sex. Model 2 is the same as model 1 plus an adjustment for chronic pancreatitis, acute pancreatitis, hepatitis B, hepatitis C, biliary disease, alcoholism, non-alcoholic fatty liver disease, income of lowest quartile, and place of residence. Model 3 is the same as model 2 plus an adjustment for the number of histories of anti-diabetic medication.

No, number; ADM, anti-diabetic medication; HR, hazard ratio; CI, confidence interval; AGI, alpha-glucosidase inhibitor.

|  | Control  (N=3,459,796) | Diabetes  (N= 864,949) | *p* value |
| --- | --- | --- | --- |
| Age (years) | 54.0 ± 12.3 | 54.0 ± 12.3 | 1 |
| 30-39 | 417,208 (12.1) | 104,303 (12.1) |  |
| 40-64 | 2,324,712 (67.2) | 581,178 (67.2) |  |
| ≥ 65 | 717,876 (20.8) | 179,469 (20.8) |  |
| Sex, male (%) | 2,277,216 (65.8) | 569,304 (65.8) | 1 |
| Body mass index (kg/m^2^) | 23.9 ± 3.0 | 25.0 ± 3.4 | <0.001 |
| Systolic BP (mmHg) | 124.3 ± 14.9 | 128.8 ± 15.6 | <0.001 |
| Diastolic BP (mmHg^)^ | 77.4 ± 9.9 | 79.9 ± 10.3 | <0.001 |
| Fasting glucose (mg/dl) | 94.1 ± 11.1 | 143.8 ± 37.8 | <0.001 |
| Total cholesterol (mg/dl) | 198.3 ± 36.1 | 204.1 ± 41.8 | <0.001 |
| Triglycerides (mg/dl) | 118.2 (118.1-118.3) | 153.7 (153.5-153.8) | <0.001 |
| HDL-C (mg/dl) | 54.3 ± 17.9 | 52.8 ± 18.6 | <0.001 |
| AST (U/l) | 25.4 (25.3-25.4) | 28.3 (28.2-28.3) | <0.001 |
| ALT (U/l) | 23.0 (22.9-23.0) | 28.5 (28.4-28.5) | <0.001 |
| GTP (U/l) | 29.6 (29.5-29.6) | 42.7 (42.7-42.8) | <0.001 |
| Smoking status (%) |  |  | <0.001 |
| Never smoker | 1,839,526 (53.2) | 432,340 (50.0) |  |
| Former smoker | 697,249 (20.2) | 164,833 (19.1) |  |
| Current smoker | 923,021 (26.7) | 267,776 (31.0) |  |
| Alcohol consumption (%) |  |  | <0.001 |
| Complete or near abstinence | 1,776,699 (51.3) | 419,941 (48.5) |  |
| Moderate (<30 g/day) | 1,387,076 (40.1) | 339,246 (39.2) |  |
| Heavy (≥30 g/day) | 296,901 (8.6) | 105,982 (12.3) |  |
| Regular exercise (%)^¶^ | 705,949 (20.4) | 165,994 (19.2) | <0.001 |
| Comorbidities |  |  |  |
| Obesity (%)^§^ | 1,162,914(33.6) | 411,165(47.5) | <0.001 |
| Hypertension (%) | 752,371 (21.8) | 304,406 (35.2) | <0.001 |
| Dyslipidemia (%) | 415,700 (12.0) | 219,692 (25.4) | <0.001 |
| Stroke (%) | 75,373 (2.2) | 27,226 (3.2) | <0.001 |
| Myocardial infarction (%) | 17,083 (0.5) | 7,457 (0.9) | <0.001 |
| TIA (%) | 25,867 (0.8) | 9,301 (1.1) | <0.001 |
| Chronic pancreatitis (%) | 4,614 (0.1) | 2,573 (0.3) | <0.001 |
| Acute pancreatitis (%) | 14,325 (0.4) | 7,118 (0.8) | <0.001 |
| Hepatitis B (%) | 64,073 (1.9) | 21,011 (2.4) | <0.001 |
| Hepatitis C (%) | 17,127 (0.5) | 8,548 (1.0) | <0.001 |
| Biliary disease (%) | 42,613 (1.2) | 16,840 (2.0) | <0.001 |
| Alcoholism (%) | 59,939 (1.7) | 34,682 (4.0) | <0.001 |
| NAFLD (%) | 330,437 (9.6) | 164,876 (19.1) | <0.001 |
| Place of residence |  |  | <0.001 |
| Urban | 1,548,758 (44.8) | 374,989 (43.4) |  |
| Rural | 1,911,038 (55.2) | 489,960 (56.7) |  |
| Income status (%) |  |  | <0.001 |
| Quartile 1 | 791,695 (22.9) | 224,867 (26.0) |  |
| Quartile 2 | 813,419 (23.5) | 221,492 (25.6) |  |
| Quartile 3 | 885,485 (25.6) | 214,792 (24.8) |  |
| Quartile 4 | 950,750 (27.5) | 196,543 (22.7) |  |
| Medical aid | 18,447 (0.5) | 7,255 (0.8) |  |
| Year of health screening exam |  |  | 1 |
| 2009 | 801,420 (23.2) | 200,355 (23.2) |  |
| 2010 | 892,428 (25.8) | 223,107 (25.8) |  |
| 2011 | 887,628 (25.7) | 221,907 (25.7) |  |
| 2012 | 878,320 (25.4) | 219,580 (25.4) |  |

**Supplementary Table S2.** Baseline characteristics of study subjects who participated in a health screening exam

Data are presented as mean ± standard deviation, or number (%).

Student’s t-test or chi-squared test was used to compare the characteristics of the study participants at baseline.

Abbreviations: BP, blood pressure; HDL-C, high-density lipoprotein-cholesterol; AST, aspartate transaminase; ALT, alanine aminotransferase; GGT, gamma-glutamyl transpeptidase; TIA, transient ischemic attack; NAFLD, non-alcoholic fatty liver disease

^§^BMI ≥25.0 kg/m^2^ according to the revised Asia-Pacific criteria of obesity, as suggested by the World Health Organization Western Pacific Region in 2000.

^¶^Regular exercise was defined according to at least three periods of vigorous- (>20 min/session) or moderate- (>30 min/session) intensity exercise per week using a self-administered and validated Korean version of the International Physical Activity Questionnaire Short Form.

|  | Number of subjects | Number of events | Duration | Incidence rate | Multivariate-adjusted HR^*^ (95% CI) | |
| --- | --- | --- | --- | --- | --- | --- |
|  |  |  |  |  | Model 1 | Model 2 |
| Total | 4,311,448 | 5,225 | 20,917,313.4 | 0.31 |  |  |
| Control | 3,451,510 | 3,794 | 13,301,161.2 | 0.29 | 1 (ref.) | 1 (ref.) |
| Diabetes | 859,938 | 1,431 | 3,296,513.8 | 0.43 | 1.54 (1.45-1.64) | 1.44 (1.32-1.57) |
| Age (years) |  |  |  |  |  |  |
| 30-39 |  |  |  |  |  |  |
| Control | 417,064 | 29 | 1,616,602.4 | 0.02 | 1 (ref.) | 1 (ref.) |
| Diabetes | 104,230 | 10 | 401,300.4 | 0.02 | 1.39 (0.68-2.86) | 2.67 (0.78-9.11) |
| 40-64 |  |  |  |  |  |  |
| Control | 2,321,886 | 1,604 | 8,941,212.4 | 0.18 | 1 (ref.) | 1 (ref.) |
| Diabetes | 579,118 | 660 | 2,223,226.2 | 0.30 | 1.66 (1.51-1.81) | 1.45 (1.28-1.65) |
| ≥ 65 |  |  |  |  |  |  |
| Control | 712,560 | 2,161 | 2,743,346.5 | 0.79 | 1 (ref.) | 1 (ref.) |
| Diabetes | 176,590 | 761 | 671,987.2 | 1.13 | 1.45 (1.33-1.57) | 1.43 (1.27-1.60) |
| Male |  |  |  |  |  |  |
| Control | 2,271,288 | 2,555 | 8,768,988.7 | 0.29 | 1 (ref.) | 1 (ref.) |
| Diabetes | 565,798 | 925 | 2,167,093.6 | 0.43 | 1.48 (1.38-1.60) | 1.36 (1.23-1.51) |
| Female |  |  |  |  |  |  |
| Control | 1,180,222 | 1,239 | 4,532,172.5 | 0.27 | 1 (ref.) | 1 (ref.) |
| Diabetes | 294,140 | 506 | 1,129,420.3 | 0.45 | 1.65 (1.49-1.83) | 1.57 (1.36-1.81) |
| Observation period (years) |  |  |  |  |  |  |
| 1.1-2.0 |  |  |  |  |  |  |
| Control | 16,878 | 808 | 8,590.5 | 94.06 | 1 (ref.) | 1 (ref.) |
| Diabetes | 7,537 | 387 | 3,774.0 | 102.55 | 1.06 (0.94-1.19) | 1.05 (0.89-1.24) |
| 2.1-4.0 |  |  |  |  |  |  |
| Control | 901,892 | 1,775 | 2,179,982.5 | 0.81 | 1 (ref.) | 1 (ref.) |
| Diabetes | 227,697 | 672 | 549,271.7 | 1.22 | 1.46 (1.34-1.60) | 1.45 (1.28-1.64) |
| ≥4.1 |  |  |  |  |  |  |
| Control | 2,532,740 | 1,211 | 11,112,588.2 | 0.11 | 1 (ref.) | 1 (ref.) |
| Diabetes | 624,704 | 372 | 2,743,468.1 | 0.14 | 1.26 (1.12-1.41) | 1.19 (1.01-1.40) |

**Supplementary Table S3.** Risk of incident pancreatic cancer in subjects who participated in a health screening exam

Cox proportional hazard models were used to estimate hazard ratios and 95% confidence intervals.

Model 1 was adjusted for age and sex. Model 2 is the same as model 1 plus an adjustment for chronic pancreatitis, acute pancreatitis, hepatitis B, hepatitis C, biliary disease, alcoholism, smoking status, non-alcoholic fatty liver disease, glucose level, body mass index, income of lowest quartile, and place of residence.

HR, hazard ratio; CI, confidence interval.

|  | Number of  subjects | Number of  events | Follow-up period | Incidence rate | Duration of diabetes | Number  of exposed  ADM | Multivariate-adjusted HR^*^ (95% CI) | | |
| --- | --- | --- | --- | --- | --- | --- | --- | --- | --- |
|  |  |  |  |  |  |  | Model 1 | Model 2 | Model 3 |
| Non-metformin | 20,991 | 103 | 84,222.2 | 1.22 | 4.0 | 1.27 | 1(ref.) | 1(ref.) | 1(ref.) |
| Metformin | 250,839 | 633 | 988,679.0 | 0.64 | 3.9 | 2.07 | 0.69 (0.56-0.86) | 0.70 (0.56-0.86) | 0.78 (0.62-0.97) |
| Non-sulfonylurea | 143,234 | 324 | 534,639.9 | 0.61 | 3.7 | 1.56 | 1(ref.) | 1(ref.) | 1(ref.) |
| Sulfonylurea | 128,596 | 412 | 538,261.3 | 0.77 | 4.2 | 2.50 | 1.29 (1.11-1.49) | 1.27 (1.09-1.47) | 1.67 (1.41-1.98) |
| Non-meglitinide | 268,882 | 726 | 1,059,764.9 | 0.69 | 3.9 | 2.00 | 1(ref.) | 1(ref.) | 1(ref.) |
| Meglitinide | 2,948 | 10 | 13,136.3 | 0.76 | 4.5 | 2.85 | 0.96 (0.51-1.79) | 0.93 (0.50-1.73) | 1.07 (0.57-2.01) |
| Non-TZD | 249,318 | 692 | 976,192.4 | 0.71 | 3.9 | 1.91 | 1(ref.) | 1(ref.) | 1(ref.) |
| TZD | 22,512 | 44 | 96,708.9 | 0.45 | 4.3 | 3.06 | 0.77 (0.57-1.05) | 0.76 (0.56-1.04) | 0.93 (0.67-1.28) |
| Non-DPP4i | 144,939 | 522 | 564,862.7 | 0.92 | 3.9 | 1.51 | 1(ref.) | 1(ref.) | 1(ref.) |
| DPP4i | 126,891 | 214 | 508,038.6 | 0.42 | 4.0 | 2.57 | 0.59 (0.51-0.70) | 0.59 (0.50-0.69) | 0.58 (0.48-0.71) |
| Non-AGI | 258,354 | 694 | 1,011,847.3 | 0.69 | 3.9 | 1.95 | 1(ref.) | 1(ref.) | 1(ref.) |
| AGI | 13,476 | 42 | 61,053.9 | 0.69 | 4.5 | 3.04 | 0.93 (0.68-1.27) | 0.90 (0.66-1.23) | 1.10 (0.79-1.52) |
| Non-insulin | 271,593 | 734 | 1,071,877.6 | 0.68 | 3.9 | 2.01 | 1(ref.) | 1(ref.) | 1(ref.) |
| Insulin | 237 | 2 | 1,023.6 | 1.95 | 4.3 | 3.03 | 3.90 (0.97-15.62) | 3.39 (0.85-13.63) | 3.92 (0.97-15.75) |

**Supplementary Table S4.** Risk of incident pancreatic cancer according to each drug exposure in subjects with more than 90-day prescription histories for more than one type of anti-diabetic medication and participated in a health screening exam

Cox proportional hazard models were used to estimate hazard ratios and 95% confidence intervals.

Model 1 was adjusted for age and sex. Model 2 is the same as model 1 plus an adjustment for chronic pancreatitis, acute pancreatitis, hepatitis B, hepatitis C, biliary disease, alcoholism, smoking status, non-alcoholic fatty liver disease, glucose level, body mass index, income of lowest quartile, and place of residence. Model 3 is the same as model 2 plus an adjustment for the number of histories of anti-diabetic medication.

No, number; ADM, anti-diabetic medication; HR, hazard ratio; CI, confidence interval; AGI, alpha-glucosidase inhibitor.

|  | Number of  subjects | Number of  events | Follow-up period | Incidence rate | Duration of diabetes | Multivariate-adjusted HR^*^ (95% CI) | | |
| --- | --- | --- | --- | --- | --- | --- | --- | --- |
|  |  |  |  |  |  | Model 1 | Model 2 |  |
| Metformin only vs. | 66,132 | 179 | 239,645.7 | 0.75 | 3.6 | 1 (ref.) | 1 (ref.) |  |
| + sulfonylurea | 111,328 | 323 | 467,711.7 | 0.70 | 4.2 | 1.09 (0.90-1.31) | 1.07 (0.89-1.30) |  |
| + meglitinide | 2,219 | 6 | 10,142.9 | 0.59 | 3.6 | 0.81 (0.36-1.83) | 0.77 (0.34-1.76) |  |
| + TZD | 20,463 | 32 | 88,124.2 | 0.36 | 3.6 | 0.64 (0.44-0.94) | 0.66 (0.45-0.97) |  |
| + DPP4i | 122,639 | 206 | 492,437.3 | 0.42 | 4.3 | 0.74 (0.60-0.90) | 0.71 (0.58-0.88) |  |
| + AGI | 11,145 | 32 | 51,275.3 | 0.62 | 3.6 | 0.92 (0.63-1.34) | 0.87 (0.59-1.28) |  |
| + insulin | 177 | 2 | 763.4 | 2.62 | 4.6 | 5.35 (1.32-21.63) | 4.09 (0.99-16.98) |  |

**Supplementary Table S5.** Risk of incident pancreatic cancer according to drug exposure in metformin users who participated in a health screening exam

Cox proportional hazard models were used to estimate hazard ratios and 95% confidence intervals.

Model 1 was adjusted for age and sex. Model 2 is the same as model 1 plus an adjustment for chronic pancreatitis, acute pancreatitis, hepatitis B, hepatitis C, biliary disease, alcoholism, smoking status, non-alcoholic fatty liver disease, glucose level, body mass index, income of lowest quartile, and place of residence.

No, number; ADM, anti-diabetic medication; HR, hazard ratio; CI, confidence interval; AGI, alpha-glucosidase inhibitor.

|  | Number of subjects | Number of events | Duration | Incidence rate | Multivariate-adjusted HR^*^ (95% CI) | |
| --- | --- | --- | --- | --- | --- | --- |
|  |  |  |  |  | Model 1 | Model 2 |
| Total | 4,885,957 | 6,538 | 14,513,945.3 | 0.45 |  |  |
| Control | 3,936,700 | 4,463 | 11,715,190.1 | 0.38 | 1 (ref.) | 1 (ref.) |
| Diabetes | 949,257 | 2,075 | 2,798,755.2 | 0.74 | 2.01 (1.91-2.12) | 1.89 (1.79-1.99) |
| Age (years) |  |  |  |  |  |  |
| 30-39 |  |  |  |  |  |  |
| Control | 321,756 | 24 | 973,288.3 | 0.02 | 1 (ref.) | 1 (ref.) |
| Diabetes | 79,420 | 22 | 239,047.4 | 0.09 | 3.74 (2.10-6.66) | 3.23 (1.702-6.13) |
| 40-64 |  |  |  |  |  |  |
| Control | 2,480,146 | 1,734 | 7,435,341.3 | 0.23 | 1 (ref.) | 1 (ref.) |
| Diabetes | 604,925 | 920 | 1,798,080.8 | 0.51 | 2.21 (2.04-2.39) | 2.04 (1.87-2.21) |
| ≥ 65 |  |  |  |  |  |  |
| Control | 1,134,798 | 2,705 | 3,306,560.4 | 0.82 | 1 (ref.) | 1 (ref.) |
| Diabetes | 264,912 | 1,133 | 761,627.0 | 1.49 | 1.85 (1.72-1.98) | 1.76 (1.64-1.90) |
| Male |  |  |  |  |  |  |
| Control | 2,186,870 | 2,693 | 6,486,130.9 | 0.42 | 1 (ref.) | 1 (ref.) |
| Diabetes | 525,590 | 1,255 | 1,540,957.3 | 0.81 | 2.03 (1.90-2.18) | 1.88 (1.75-2.02) |
| Female |  |  |  |  |  |  |
| Control | 1,749,830 | 1,770 | 5,229,059.2 | 0.34 | 1 (ref.) | 1 (ref.) |
| Diabetes | 423,667 | 820 | 1,257,797.9 | 0.65 | 1.98 (1.82-2.15) | 1.89 (1.74-2.06) |

**Supplementary Table S6.** Risk of incident pancreatic cancer in subjects after excluding the subjects whose follow-up period less than two years.

Cox proportional hazard models were used to estimate hazard ratios and 95% confidence intervals.

Model 1 was adjusted for age and sex. Model 2 is the same as model 1 plus an adjustment for chronic pancreatitis, acute pancreatitis, hepatitis B, hepatitis C, biliary disease, alcoholism, non-alcoholic fatty liver disease, income of lowest quartile, and place of residence.

HR, hazard ratio; CI, confidence interval.
